# First Evidence of In Vitro Effects of C6O4—A Substitute of PFOA—On Haemocytes of the Clam *Ruditapes philippinarum*

**DOI:** 10.3390/toxics9080191

**Published:** 2021-08-19

**Authors:** Jacopo Fabrello, Francesca Targhetta, Maria Ciscato, Davide Asnicar, Ilaria Bernardini, Massimo Milan, Tomaso Patarnello, Maria Gabriella Marin, Valerio Matozzo

**Affiliations:** 1 Department of Biology, University of Padova, Via Ugo Bassi 58/B, 35131 Padova, Italy; jacopofabrello@gmail.com (J.F.); francesca.targhetta.1@studenti.unipd.it (F.T.); cisc95@gmail.com (M.C.); davide.asnicar@gmail.com (D.A.); mgmar@mail.bio.unipd.it (M.G.M.); 2 Department of Comparative Biomedicine and Food Science, University of Padova, Viale dell’Università 16, 35020 Legnaro, Italy; ilaria.bernardini.1@phd.unipd.it (I.B.); massimo.milan@unipd.it (M.M.); tomaso.patarnello@unipd.it (T.P.)

**Keywords:** C6O4, per- and poly-fluoroalkyl substances, bivalve, haemocyte, in vitro assays

## Abstract

Alternative chemicals to per- and poly-fluoroalkyl substances have recently been introduced in various industrial processes. C6O4 (difluoro{[2,2,4,5-tetrafluoro-5-(trifluoromethoxy)-1,3-dioxolan-4-yl]oxy}acetic acid) is a new surfactant and emulsifier used as a replacement for perfluorooctanoic acid (PFOA). From an ecotoxicological point of view, in vitro assays are useful tools for assessing the negative effects and understanding the mechanisms of action of chemicals at the cellular level. Here, we present the results of an in vitro study in which the effects of C6O4 were evaluated—for the first time—on haemocytes of the clam *Ruditapes philippinarum*. Cells were exposed to three concentrations of C6O4 (0.05, 0.5, 5 μg/mL) and the effects on haemocyte viability, haemocyte morphology, differential haemocyte count, lysosomal membrane stability, superoxide anion production, acid phosphatase, and β-glucuronidase activities, as well as on the percentage of micronuclei and chromosomal aberrations were evaluated. The results demonstrated that C6O4 significantly affected haemocyte morphology, lysosomal membrane stability, hydrolytic enzyme activity, and superoxide anion production, and promoted chromosomal aberrations. To the best of our knowledge, this is the first study revealing the in vitro effects of C6O4, a substitute for PFOA, on haemocytes from a bivalve species.

## 1. Introduction

Per- and polyfluoroalkyl substances (PFAS) are classified as persistent organic pollutants (POPs) due to their high resistance to degradation [1]. However, they are used in many industrial processes, such as the fluoropolymer industry, the textile and food packaging industry, aqueous film-forming foam, and metal-plating industries [2,3].

One of the mainly used PFAS is perfluorooctanoic acid (PFOA), which has been recently included, along with its salts, into Annex A of the Stockholm Convention [4]. PFOA has been used as a wetting agent in polytetrafluoroethylene (PTFE) production [5], but since the 2000s, the primary chemical U.S. industries have started to phase it out [6]. Indeed, industries started to replace long-chain PFAS (including PFOA), which are persistent and can be bioaccumulated, with shorter compounds. However, short-chain PFAS have lower technical performance, so higher quantities are generally used [7].

**Figure 1:**
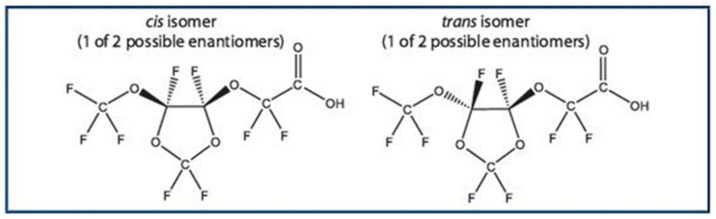
A single enantiomer of each *cis/trans* isomer of C6O4.

Perfluoroalkyl ether carboxylic acids (PFECAs) are a relatively new class of short-chain PFAS used to replace PFOA [8]. Some of these compounds, such as GenX, HPFO-TA, and ADONA, have recently been detected both in freshwater and seawater ecosystems [9,10,11,12,13]. One of the main substitutes of PFOA is C6O4, also named (difluoro{[2,2,4,5-tetrafluoro-5-(trifluoromethoxy)-1,3-dioxolan-4-yl]oxy}acetic acid) (Figure 1).

It is classified as perfluoro ether non-polymer with saturated bonds, and dozens of tons are produced every year [14,15,16]. It is used as a surfactant and emulsifier in polymerization processes of other substances (polymers) and the manufacturing of cast film, fittings, valves, tapes, and anti-stick coating (food contact materials) [15]. C6O4 has been recorded at a maximum concentration of 345 ng/L in superficial Italian waters [17], both in the Po River and in its tributaries (290 ng/L) [15,17]. In addition, a C6O4 concentration up to 3265 ng/L was found in groundwaters downstream of a fluorochemical factory [17].

Despite the evidence of the presence of C6O4 in aquatic ecosystems, to our knowledge, only a few studies have investigated the negative effects of the compound in bivalve molluscs [18]. To provide the first data about C6O4 cytotoxicity in bivalves, an in vitro approach was used to evaluate the effects of the compound on *Ruditapes philippinarum* haemocytes. It is well-known that haemocytes are involved in immune responses in bivalves [19,20]. The hypothesis we tested was that in vitro exposure to C6O4 affects the morpho-functional features of clam haemocytes.

## 2. Materials and Methods

### 2.1. Clams, Haemolymph Collection, and Haemocyte Exposure

Specimens of *R. philippinarum* were collected from a reference site inside a licensed area for clam culture in the southern basin of the Lagoon of Venice (Italy). Prior to the experiments, clams were acclimatised for 7 days in large aquaria containing a sandy bottom and aerated seawater (salinity of 35 ± 1, temperature of 16 ± 0.5 °C) and fed daily ad libitum with a microalgae mixture (*Isochrysis galbana, Phaeodactylum tricornutum,* and *Tetraselmis chui*).

For each experiment, three different pools of haemolymph from four clams each were used. The haemolymph was collected from the adductor muscles with a plastic syringe, stored on ice, and added to an equal volume of 0.38% sodium citrate in 0.45 µm of filtered seawater (FSW), pH 7.5, to prevent clotting. The haemolymph was then centrifuged at 800× *g* for 10 min and haemocytes were resuspended in FSW to prepare short-term haemocyte cultures. To this aim, the method of Ballarin et al. [21] was used. Briefly, 60 μL of haemocyte suspension (at a final concentration of 10^6^ cells/mL) was placed in culture chambers made by a Teflon ring (internal diameter of 15 mm and 1 mm thick) smeared with petroleum jelly, glued to a siliconised glass slide, and covered with a coverslip. Chambers were kept upside down for 30 min at room temperature to allow the haemocytes to settle and adhere to the coverslips.

The stock solution of C6O4 (50 μg/mL) was provided in methanol (Wellington Laboratories, Guelph, Ontario, Canada). The following exposure concentrations were tested: 0 (1st control), 0+methanol (2nd control), 0.05, 0.5, and 5 μg/mL of FSW. In the 1st control, only FSW was added, whereas, in the 2nd control, methanol was added at the same concentration present in the highest dose of C6O4 tested, namely 5 μg/mL. The three concentrations tested are representative of a possible environmental level, an intermediate level, and a level that could be reached in a worst-case scenario of continuous release of the compound into the aquatic environments.

After the adhesion of haemocytes to the coverslips, FSW was removed from the culture chambers and replaced with an equal volume of FSW (control), or 5 μg/mL of methanol in FSW, or three concentrations of C6O4 (0.05, 0.5, and 5 μg/mL in FSW). Cells were exposed for 30 min at room temperature. FSW, methanol, and C6O4 solutions were then removed from the culture chambers and the haemocytes were processed according to each experimental procedure described below.

Each experiment was repeated three times, using three pools of haemolymph from three different pools of clams each time. Two chambers (= two slides) for each experimental condition were prepared and 100 haemocytes per slide were observed under a light microscope (LM).

### 2.2. Haemocyte Viability Assay

After exposure to C6O4, the haemocytes were incubated with 0.25% Trypan Blue in FSW for 5 min at room temperature and observed in vivo under LM at 1000X. The number of haemocytes that were stained blue was then calculated.

### 2.3. Neutral Red Retention Assay

Lysosomal membrane stability was assessed following the method of Lowe et al. [22], modified. The stock solution of Neutral Red (NR) (0.4%) was prepared in FSW, whereas the working solution was obtained by diluting 10 µL of the stock solution in 5 mL of FSW. After exposure of the haemocytes, 60 µL of NR working solution was added to each chamber. After 10 min, the haemocytes were observed in vivo under the LM at 1000X. The lysosomal stability index was expressed as the percentage of haemocytes showing dye loss from lysosomes into the cytosol, which appeared reddish-pink.

### 2.4. Haemocyte Morphology

Treated and untreated haemocytes were fixed for 30 min at 4 °C (1% glutaraldehyde and 1% sucrose in FSW), washed in phosphate-buffered saline (PBS: 1.37 M NaCl, 0.03 M KCl, 0.015 M KH_2_PO_4_, 0.065 M Na_2_HPO_4_, pH 7.2) for 10 min, stained for 10 min in 10% Giemsa solution, washed in distilled water, and mounted on glass slides with Aquovitrex (Carlo Erba). The slides were then observed under the LM at 1000X. The percentage of stained haemocytes with a round shape was then estimated.

### 2.5. Differential Cell Count (Pappenheim’s Panoptical Staining)

After exposure, the haemocytes were fixed as described above, stained for 3 min in May-Grünwald’s dye (Fluka), washed in distilled water, stained for 5 min in 5% Giemsa, washed in distilled water, and mounted. The percentage of haemocytes with basophil (blue) or acidophils (dark pink) granules was calculated under the LM.

### 2.6. Hydrolytic Enzymes

After exposure to C6O4, the haemocytes were fixed as described previously and incubated in reaction mixtures to reveal enzyme activities.

β-glucuronidase: Fixed haemocytes were washed for 10 min in 0.1 M sodium acetate buffer (pH 5.2) and incubated for 2 h at 37 °C with a mixture containing 4 mg naphthol AS-BI β-glucuronide (Sigma) dissolved in 250 μL dimethylformamide (DMF), 400 μL solution A (0.4 g pararosaniline (Fluka), 2 mL HCl 37%, 8 mL distilled water), 400 μL solution B (4% NaNO_2_ in distilled water) and 20 mL of 0.1 M sodium acetate buffer (pH 5.2) [23]. The haemocytes were then washed for 10 min in sodium acetate buffer and mounted. Positive haemocytes were stained red.

Acid phosphatase: Fixed haemocytes were washed for 10 min in 0.1 M sodium acetate buffer, pH 5.2, and then incubated for 1 h at 37 °C in a mixture similar to that described above but containing 10 mg naphthol AS-BI phosphate (Sigma) previously dissolved in 400 μL DMF as a substrate [24]. Positive haemocytes were stained red.

### 2.7. Intracellular Superoxide Anion Detection

Intracellular O_2_- was detected according to the method of Song and Hsieh [25]. Both untreated and treated haemocytes were incubated for 60 min with 0.2% nitro blue tetrazolium (NBT) in FSW, fixed as described above and washed for 10 min in PBS. The reaction product (formazan) was solubilised by adding a solution containing 120 μL of 2 M KOH and 140 μL of DMSO for 30 min. The slides were then mounted as described above. The granules of precipitated formazan appeared in blue.

### 2.8. Micronuclei (MN) Assay

The MN test was performed according to the method of Pavlica et al. [26]. After haemolymph sampling, the pooled haemolymph (500 µL) was centrifuged at 800× *g* for 10 min and haemocytes were resuspended in FSW, methanol, or C6O4 concentrations. The haemocytes were incubated for 30 and 60 min at room temperature. Thereafter, 100 µL of treated and untreated haemocytes were put on a slide (2 slides per experimental conditions) and left at room temperature for 15 min in a humidified chamber to allow the haemocytes to adhere. The haemocytes were then fixed in a solution of glutaraldehyde (25% in 0.45 µm of filtered seawater) for 5 min. The slides were washed with phosphate-buffered saline (PBS), stained with Hoechst dye solution (1 µg/mL) for 5 min, washed again, mounted in glycerol–McIlvaine buffer (1:1), and kept at 4 °C in the dark. The slides were observed under an Olympus CX31 fluorescent microscope. Two hundred nuclei were counted for each slide. Micronuclei and chromosome aberrations (e.g., multipolar, or multinucleated cells, eight-shaped nuclei) were identified according to Kirsch-Volders et al. [27] and Pavlica et al. [26], and the results were expressed as the MN frequency (MN‰) and aberration frequency.

### 2.9. Statistical Analysis

The results were checked for normal distribution (Shapiro–Wilk test) and homogeneity of the variance (Bartlett test). The data were statistically compared using a one-way ANOVA test, followed by Fisher’s Least Significant Difference (LSD) for pairwise comparisons. The results from the MN assay were statistically compared by a two-way ANOVA test, with exposure time and treatment as variables and biomarkers as cases. The LSD post-hoc test was used to evaluate significant differences among the experimental groups. Each experiment was performed in triplicate (*n* = 3). The results are expressed as the mean ± standard deviation (SD). The software package Statistica 13.4 (TIBCO Software Inc.) was used for the statistical analyses.

## 3. Results

**Figure 2:**
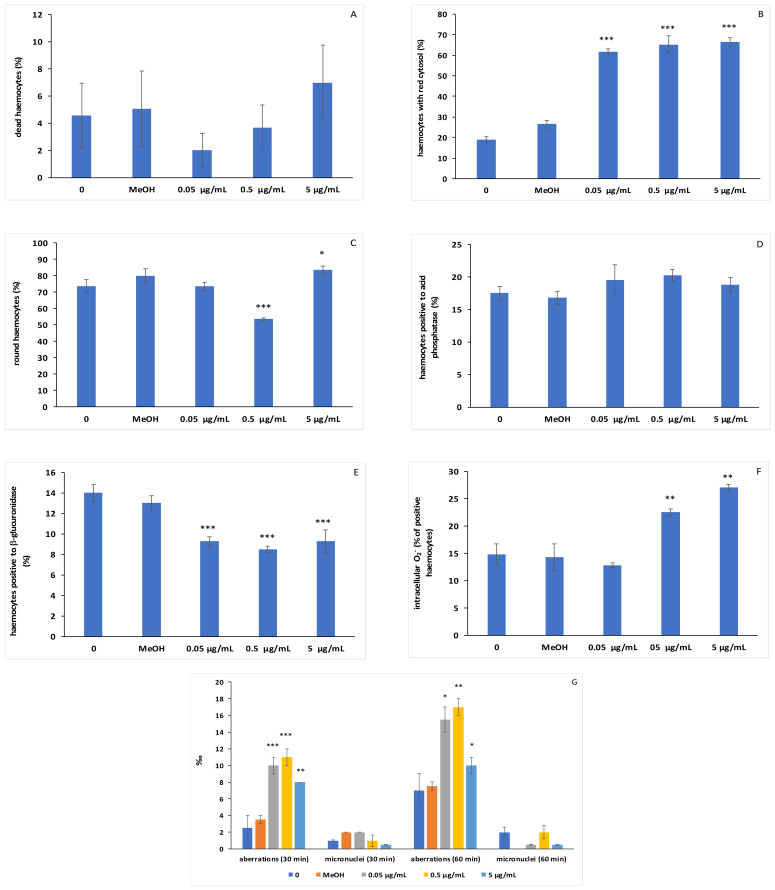
Haemocytes of *R. philippinarum* exposed to C6O4. (**A**) Percentage of dead haemocytes stained with Trypan Blue. (**B**) Percentage of haemocytes showing Neutral Red loss into the cytoplasm. (**C**) Percentage of round-shaped haemocytes stained with Giemsa. (**D**) Percentage of haemocytes positive to acid phosphate. (**E**) Percentage of haemocytes positive to β-glucuronidase. (**F**) Percentage of haemocytes with blue precipitated formazan, denoting O_2_- production. (**G**) Frequency of chromosomal aberrations and micronuclei after 30 and 60 min of exposure of haemocytes to C6O4. Values are means + SD, *n* = 3. Asterisks denote significant differences with respect to controls (0): * *p* < 0.05, ** *p* < 0.01, *** *p* < 0.001.

**Figure 3:**
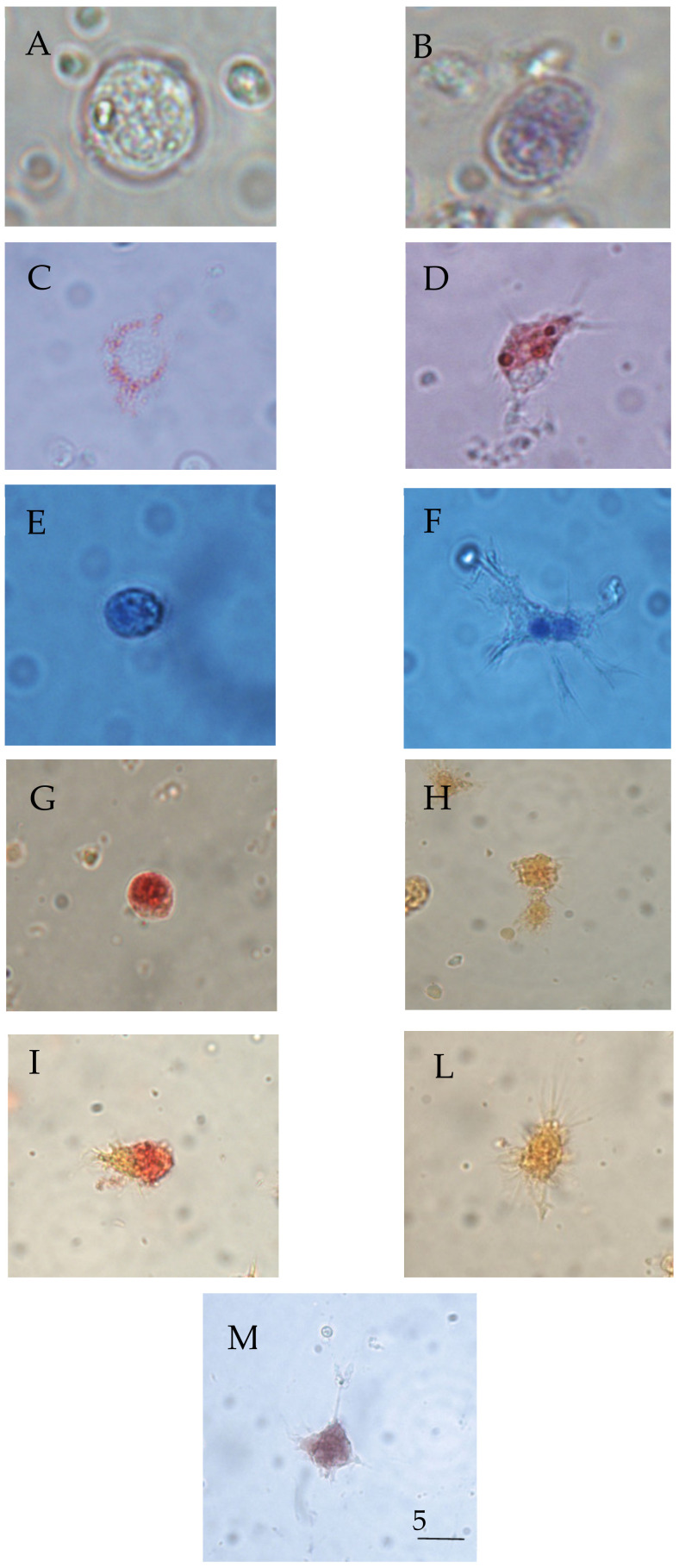
Light micrographs of *R. philippinarum* haemocytes following exposure to C6O4. (**A**,**B**) Unfixed cells stained with Trypan Blue (live cell in (**A**), dead cell in (**B**)). (**C**,**D**) Haemocytes vitally stained with Neutral Red (intact cell with evident lysosomes in (**C**), damage cell showing dye loss into the cytosol in (**D**)). (**E**,**F**) Haemocytes stained with Giemsa’s dye (round cell in (**E**), spreading cell in (**F**)). (**G**) Haemocyte positive to acid phosphatase. (**H**) Haemocyte not positive to acid phosphatase. (**I**) Haemocyte positive to β-glucuronidase. (**L**) Haemocyte not positive to β-glucuronidase. (**M**) Haemocyte with deposits of formazan (blue) after solubilisation with KOH/DMSO solution. Bar length: 5 µm.

In vitro exposure to C6O4 did not significantly affect (ANOVA: F = 0.69, *p* = 0.61) haemocyte viability (Figure 2A). Indeed, the percentage of dead haemocytes stained blue (Figure 3A,B) did not exceed 8% at all the concentrations tested, including the controls. The results indicate that the concentrations tested were sublethal for haemocytes.

This study demonstrated that C6O4 significantly (ANOVA: F = 83.23, *p* = 0.000) affected the lysosomal membrane stability of haemocytes. In detail, the LSD post-hoc test revealed a significant (*p* < 0.001) increase in the percentage of haemocytes showing dye loss from lysosomes into the cytosol when compared with the controls (Figure 2B). Consequently, the haemocytes appeared reddish-pink after treatment with all the concentrations of C6O4 tested (Figure 3C,D).

Exposure to C6O4 significantly affected (ANOVA: F = 14.53, *p* = 0.000) haemocyte morphology. Indeed, the percentages of round-shaped haemocytes (Figure 3E,F) decreased significantly (*p* < 0.001) at 0.5 µg/mL but increased significantly (*p* < 0.05) at 5 µg/mL (Figure 2C) when compared to the controls.

Conversely, exposure to the contaminant did not significantly affect the proportion of acidophil (ANOVA: F = 0.58, *p* = 0.67) and basophil (F = 0.59, *p* = 0.68) haemocytes among the experimental groups (data not shown).

As for hydrolytic enzyme activities, ANOVA demonstrated that 30 min of incubation with C6O4 was unable to affect the percentage of haemocytes positive to acid phosphatase (F = 1.03, *p* = 0.42) (Figure 2D and Figure 3G,H), whereas in vitro exposure significantly (ANOVA: F = 9.18, *p* = 0.000) influenced β-glucuronidase activity, with a significant (LSD test: *p* < 0.001) decrease in the number of positive cells to the enzyme at all the concentrations tested compared to the controls (Figure 2E and Figure 3I–L).

The in vitro treatment with C6O4 significantly (ANOVA: F = 16.25, *p* = 0.000) affected the production of intracellular O_2_- in haemocytes. In detail, the percentage of cells with blue granules of precipitated formazan increased significantly (*p* < 0.01) at 0.5 and 5 μg/mL, with respect to the controls (Figure 2F and Figure 3M).

**Figure 4:**
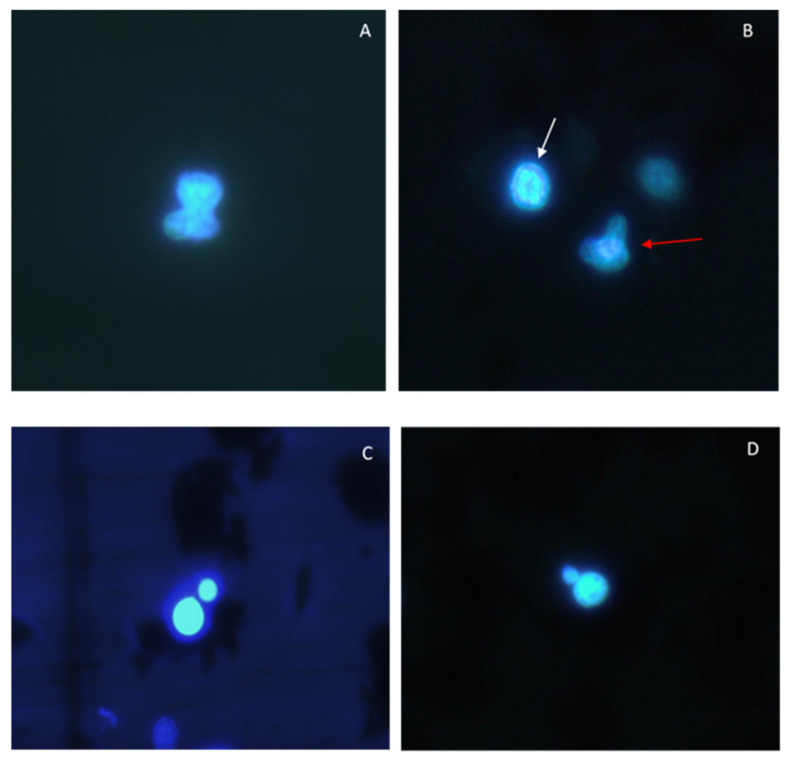
Chromosomal aberrations and micronuclei in *R. philippinarum* haemocytes following exposure to C6O4. (**A**) Eight-shaped nucleus. (**B**) Chromatin margination toward the nuclear membrane (white arrow) and multipolar cell (red arrow). (**C**,**D**) Micronuclei.

Lastly, significant increases in the frequency of chromosomal aberrations (Figure 2G and Figure 4A,B) were observed in haemocytes treated for 30 and 60 min with all the concentrations of C6O4. Conversely, exposure to the compound did not induce increments in MN frequency (Figure 2G and Figure 4C,D).

## 4. Discussion

In vitro tests are inexpensive, have high reproducibility, and are useful tools for evaluating both the negative effects and mechanisms of action of environmental contaminants [28]. In this study, we evaluated, for the first time, the in vitro effects of C6O4 on haemocytes of the clam *R. philippinarum*, which play an important role in immune defences [19,20].

Exposure of haemocytes to C6O4 did not significantly affect cell viability, the percentage of haemocytes stained blue (= dead cells) being less than 10% at all the concentrations tested, including the controls. Consequently, all concentrations of C6O4 tested in this study were sublethal. To our knowledge, no information concerning the lethal effects of C6O4 on haemocytes from aquatic invertebrates is available in the literature. As for other perfluoroalkyl substances (PFAS), an in vivo study demonstrated that exposure for 7 days of the green mussels *Perna viridis* to perfluorooctane sulfonate (PFOS), PFOA, perfluorononanoic acid (PFNA), and perfluorodecanoic acid (PFDA) significantly decreased cell viability, starting from a concentration of 100 µg/L [29]. A dose-dependent decrease in cell viability was also observed in primary cultured hepatocytes of freshwater tilapia (*Oreochromis niloticus*) treated for 24 h with PFOS or PFOA (1 to 30 mg/L) [30]. As for other cell models, FRTL5 rat thyroid cell lines and normal human thyroid cells (NHTs) were treated for 24, 48, 72, and 144 h with increasing concentrations (0, 0.01, 0.1, 1, 10, and 100 ng/mL) of C6O4, PFOS, and PFOA, and cell viability was then evaluated [31]. Exposure to C6O4 did not affect FRTL5 and NHT cell viability. Conversely, PFOS reduced the cell viability of FRTL5 rat thyroid cells and NHTs, whereas PFOA reduced the cell viability of FRTL5 only [31]. Both cytotoxic and genotoxic effects of PFOA and PFOS on human HepG2 cells were investigated after 1 or 24 h of exposure [32]. Similar to what was observed in our study, short-term exposure of cells for 1 h to both the compounds did not reduce cell viability. Conversely, long-term exposure (24 h) of human HepG2 cells induced a significant decrease in cell viability after treatment with PFOS (300, 400, and 600 μM) and PFOA (200, 400, 600, and 800 μM) [32]. Considering such controversial results, more efforts should be done to investigate in depth the cytotoxic effects of C6O4 or other PFAS in differing cell models.

A significant increase in the percentage of haemocytes with reddish-pink cytosol following in vivo staining with NR was observed at all the tested concentrations, suggesting that C6O4 was able to induce a marked destabilisation of lysosomal membranes. Conversely, lysosomes of control haemocytes retained NR after its uptake (only a small fraction of control haemocytes showed a red cytosol). Similar results have been obtained after in vivo exposure of mussels to PFAS. A significant reduction in NR retention time was recorded after exposure of animals to 1000 µg/L of all PFAS, indicating a reduction of lysosome membrane stability [29]. We speculated that exposure to C6O4 could affect Mg^2+^-ATPase, a proton pump of lysosomal membranes that maintains the internal acidic environment of lysosomes [33]. The malfunctioning of such ATPase can allow the passage of lysosomal contents, including NR, into the cytosol [22]. As for alternative explanations, Nobels et al. [34], using a multiple endpoint bacterial reporter assay, demonstrated that PFOA and PFNA induced higher levels of membrane damage than PFOS. The authors suggested that membrane damage was probably due to increased membrane fluidity caused by the detergent-like nature of the compounds [34]. In in vitro cell-based assays, PFOS was shown to increase membrane fluidity in fish leukocytes (analysed by flow cytometry) at 33 and 100 µM (equivalent to 16.5 and 50 mg/L) [35]. The authors suggested that increased membrane fluidity following exposure to PFOS could be a consequence of decreases in the cholesterol content of the membranes, resulting in further increases in membrane fluidity [35]. In the present study, a negative effect of reactive oxygen species (ROS) in destabilizing lysosomal membranes cannot be excluded, since superoxide anion levels increased in the haemocytes exposed to the contaminant (see below for discussion).

The results of this study indicate that exposure for 30 min of clam haemocytes to C6O4 affected the cell morphology. Indeed, the percentage of rounded cells first decreased at 0.5 and then increased at 5 µg/mL. In *R. philippinarum*, both round and spreading cells circulate in the haemolymph [19]. The increased number of round cells was probably due to a negative effect of C6O4 on the cytoskeleton organisation of haemocytes that withdrew pseudopodia, assuming a spherical shape. Conversely, no morphological differences were recorded in human HepG2 cells incubated for 1 h with PFOS and PFOA, whereas intracellular vacuoles in several cells, a disappearance of the extracellular matrix, and a substantial number of cells suspended in culture medium were detected after 24 h of exposure, starting from a concentration of 200 μM of PFOA and 300 μM of PFOS. In that study, only a few adherent cells were rounded, but a significant increase in suspended cells and cell debris was found at higher concentrations of PFOA (more than 600 μM) and PFOS (400 μM) [32]. In any case, further studies are necessary to clarify better the possible relationship between exposure to C6O4 and morphological alterations in haemocytes from clams, as no linear results were observed at the two higher concentrations tested.

In *R. philippinarum*, three different subpopulations of haemocytes have been detected, namely basophils (50%), neutrophils (47%), and acidophils (3%) [19]. In this study, we assessed the ability of C6O4 to alter the proportion of haemocyte subpopulations in *R. philippinarum*. Based on the results obtained, we can rule out this possibility, at least under the experimental conditions tested in our study.

In addition, Cima et al. [19] demonstrated that haemocytes of *R. philippinarum* possess a battery of hydrolytic enzymes that are stored in lysosomes. In molluscs, such enzymes play a key role in destroying engulfed materials [36]. Consequently, in the present study, we evaluated the effects of C6O4 on the activity of two important enzymes, β-glucuronidase and acid phosphatase, estimating the percentage of haemocytes that produce the two enzymes, as described previously [37]. Interestingly, two different patterns of variations were observed in this study. Indeed, the percentage of haemocytes with active acidic phosphatase did not change significantly following exposure to C6O4, whereas the percentage of cells producing β-glucuronidase decreased significantly in treated haemocytes. Based on the results obtained, we can hypothesize an effect of C6O4 on the lysosomes, as also suggested by the results of the NR assay. However, the different response of the two enzymes suggests further studies to better clarify the mode of action of C6O4 on the lysosomal compartment.

In cells, oxidative stress may occur when ROS production exceeds the antioxidant defence system, causing the peroxidation of membrane lipids, damage to DNA, proteins, and enzymes, and the activation of apoptotic and/or necrotic processes [38]. Oxidative stress can be promoted by exposure of the organisms to a variety of environmental contaminants, including PFAS [39,40]. In this study, we demonstrated that in vitro exposure of clam haemocytes to C6O4 increased the percentage of cells with blue granules of precipitated formazan, which indicated O_2_- production. In our opinion, this is an important result because it could explain one of the possible mechanisms of action of C6O4, which is promoting oxidative stress. Interestingly, increased ROS production has previously been observed in several in vitro and in vivo studies following the exposure of differing cell models and various species to PFAS. For example, a significant induction of ROS was recorded in primary cultured hepatocytes of the freshwater tilapia *O. niloticus* exposed for 24 h to PFOS or PFOA (0, 1, 5, 15, and 30 mg/L) [30]. In embryos of zebrafish (*Danio rerio*), increased ROS production was observed after exposure to PFOS and PFNA [41,42,43,44]. Wielsøe et al. [39] demonstrated that all the PFAS tested (except for perfluorododecanoate) significantly increased intracellular ROS generation in a human hepatoma cell line (HepG2) exposed in vitro for 24 h to different concentrations (from 2 × 10^−7^ to 2 × 10^−5^ M). In the same cell line, Eriksen et al. [45] demonstrated that PFOA and PFOS, but not perfluorobutane sulfonate (PFBS) and perfluorohexanoic acid (PFHxA), increased the intracellular ROS production by 1.52-fold and 1.25-fold, respectively. Conversely, neither PFOS nor PFOA induced a significant increase in ROS generation in human cell line HepG2 [32]. Overall, both the results of the present study and most of those available in the literature suggest that one of the possible effects of PFAS, including C6O4, is ROS-mediated cytotoxicity.

Micronuclei induction is a biomarker commonly used to detect the genotoxicity of pollutants to aquatic organisms [46,47,48]. In this study, no significant increases in MN frequency were observed in the haemocytes exposed to C6O4. Considering all the experimental conditions tested, the MN frequency did not exceed 2‰. Conversely, significant increases in chromosomal aberrations, such as nuclear fragmentation, multipolar or multinucleated cells, eight-shaped nuclei, and chromatin margination toward the nuclear membrane were detected in C6O4-treated haemocytes. Interestingly, eight-shaped nuclei were detected in agranular haemocytes from mussels (*Mytilus galloprovincialis*) exposed for 2 days to benzo[a]pyrene (0, 5, 50, 100, 500, 1000 ppb) [49], while chromatin margination forming dense crescent-shaped aggregates by the nuclear membrane was observed in haemocytes from great ramshorn snail, *Planorbarius corneus*, exposed for 14 days to pentachlorophenol (10 to 450 µg/L) [26]. As for PFAS, in vivo exposure for 7 days of the green mussel *P. viridis* to various concentrations (0.1, 1, 10, 100, and 1000 μg/L) of PFOS, PFOA, PFNA, and PFDA caused a significant increase in MN frequency of haemocytes, at least at the two highest concentrations tested [50]. In the Japanese medaka *Oryzias latipes*, time- and concentration-dependent increases in both MN frequency and cell abnormalities (fragmented apoptotic cells, lobed nuclei, and bean-shaped cells) were observed in erythrocytes after the exposure of fish for 28 days to 0.1, 0.5, and 2.5 mg/L of PFDA [51]. As for other cell lines, in vitro exposure for 72 h of human hepatocarcinoma cell line HepG2 to PFECAs did neither cause mutagenic nor clastogenic effects [52]. In the same cell line, Florentin et al. [32] demonstrated that neither PFOS nor PFOA was able to induce an increase in micronucleus frequency. Overall, our study demonstrated that in vitro exposure of clam haemocytes for 30 or 60 min to C6O4 did not induce MN formation. However, significant increases in cell abnormalities observed in C6O4-treated haemocytes should be carefully considered, as they may indicate a potential genotoxic effect of the compound on bivalve haemocytes. This aspect needs to be more fully investigated both in vitro and in vivo studies.

## 5. Conclusions

The results of this study demonstrate that C6O4 can affect the morpho-functional features of haemocytes from *R. philippinarum*. The ability of the compound to increase intracellular ROS production, which, in turn, could cause damage to macromolecules, such as lipids, proteins, and DNA, is noteworthy. In this regard, increases in the frequency of cell/chromosomal abnormalities are suspected to be a consequence of the cytotoxic potential of C6O4. In any case, further studies are necessary to investigate the mechanisms of action of C6O4 in *R. philippinarum* in depth, as well as those in other bivalve species.

## Data Availability

Not applicable.
